# Enzyme Mimicking Based on the Natural Melanin Particles from Human Hair

**DOI:** 10.1016/j.isci.2019.100778

**Published:** 2019-12-18

**Authors:** Sheng Hong, Qiu-Ling Zhang, Di-Wei Zheng, Cheng Zhang, Yu Zhang, Jing-Jie Ye, Han Cheng, Xian-Zheng Zhang

**Affiliations:** 1 Key Laboratory of Biomedical Polymers of Ministry of Education & Department of Chemistry, Wuhan University, Wuhan 430072, P. R. China

**Keywords:** Biochemistry, Natural Product Biochemistry, Materials Science, Biomaterials

## Abstract

Natural enzymes are mainly composed by the protein part and metallic cofactor part, both of which work cooperatively to achieve high catalytic activity. Here, natural melanin particles (NMPs) were extracted from human hair and further bound with metal ions to mimic natural enzymes. The different metal-bound NMPs (M-NMPs) exhibited different enzyme-like activities with great promise in diverse biomedical applications. It was found that Fe-bound NMPs (Fe-NMPs) showed outstanding peroxidase (POD)-like activity that possessed potential in antibacterial applications, and Mn-bound NMPs (Mn-NMPs) displayed catalase (CAT)-like activity with a remarkable radiotherapy sensitization effect in cancer therapy. Besides, Cu-bound NMPs (Cu-NMPs) could serve as combined POD, superoxide dismutase (SOD), and CAT alternatives, which exhibited prominent reactive oxygen species (ROS) scavenging ability, revealing great potential in anti-inflammation. The versatile enzyme-like activities of M-NMPs derived from hair might give extensive perspective for designing biomedical materials and provide a promising tool in solving biomedical problems.

## Highlights

• Natural enzyme mimicking based on human hairs
• Efficient, stable, and low-cost enzyme alternatives
• Different enzyme-like activities of different M-NMPs
• Enzyme-like activities provide the potential in biomedical application

## Graphical Abstract

**Figure:**
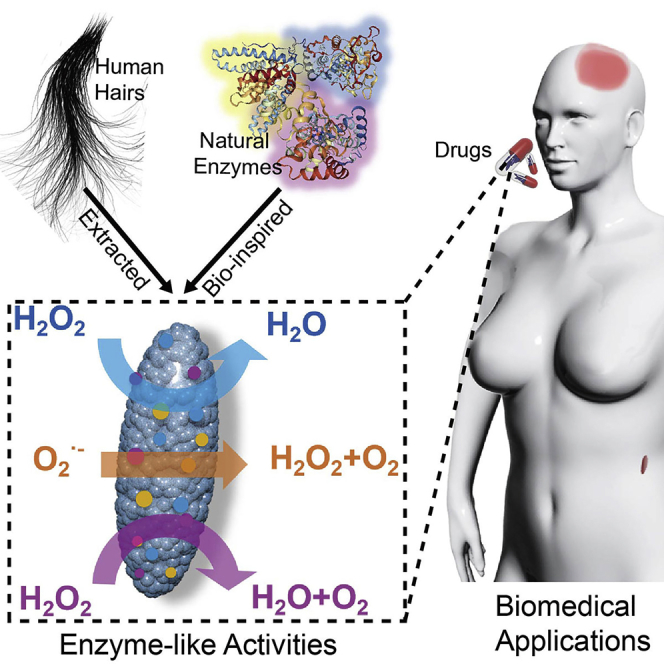

## Introduction

Natural enzymes, playing crucial roles in all the life activities, have been extensively applied in various fields owing to their superior performance (Chen et al., 2018, Li et al., 2017a, Triplett et al., 2018, Wu et al., 2019, Yang et al., 2018). However, intrinsic drawbacks of natural enzymes such as high cost and low stability seriously limit their practical applications. To overcome the disadvantages, great efforts have been made to develop natural enzyme alternatives with high performance (Wei and Wang, 2013, Yang et al., 2019). In the past few decades, varieties of bio-inspired nanomaterials have been proposed to harbor enzyme-like activities for serving as high-performance natural enzyme alternatives (Wang et al., 2016, Wang et al., 2018). For example, MnO_2_ and CeO_2_ nanoparticles have been reported to possess both catalase (CAT)-like and superoxide dismutase (SOD)-like catalytic activities that can protect cells against oxidative damages through eliminating harmful reactive oxygen species (ROS) (Kim et al., 2012, Li et al., 2017b). In addition, Fe_3_O_4_ nanoparticles also exhibited peroxidase (POD)-like activity for immunohistochemical detection (Fan et al., 2012, Gao et al., 2007). Although these enzyme alternatives showed considerable potential applications in biomedicine, the low efficiency and unsatisfied selectivity still restrict their practical applications.

In general, natural enzymes are mainly consisting of the protein part and metallic cofactor part. The protein part, namely the peptide chain containing functional groups such as amino, carboxyl, hydroxy, and indolyl groups, could adsorb the substrate and provide active sites for the convenience of substrate binding. And the metallic cofactor part is generally metal ion or the metal complex, playing an important role in electron transmission. Both of them are indispensable for working together to achieve the catalytic activity of enzymes (Zastrow et al., 2011, Lu et al., 2009). For example, POD, a kind of protein containing iron porphyrin as cofactor, could catalyze the oxidation of substrate by hydrogen peroxide as electron acceptor (Das and Hecht, 2007). Cu and Mn were also found to act as the active center in SOD, an enzyme disproportionation of superoxide anion, which could serve as a significant antioxidant enzyme in nearly all living cells (Robinson and Winge, 2010, Huang et al., 2000).

**Scheme 1:**
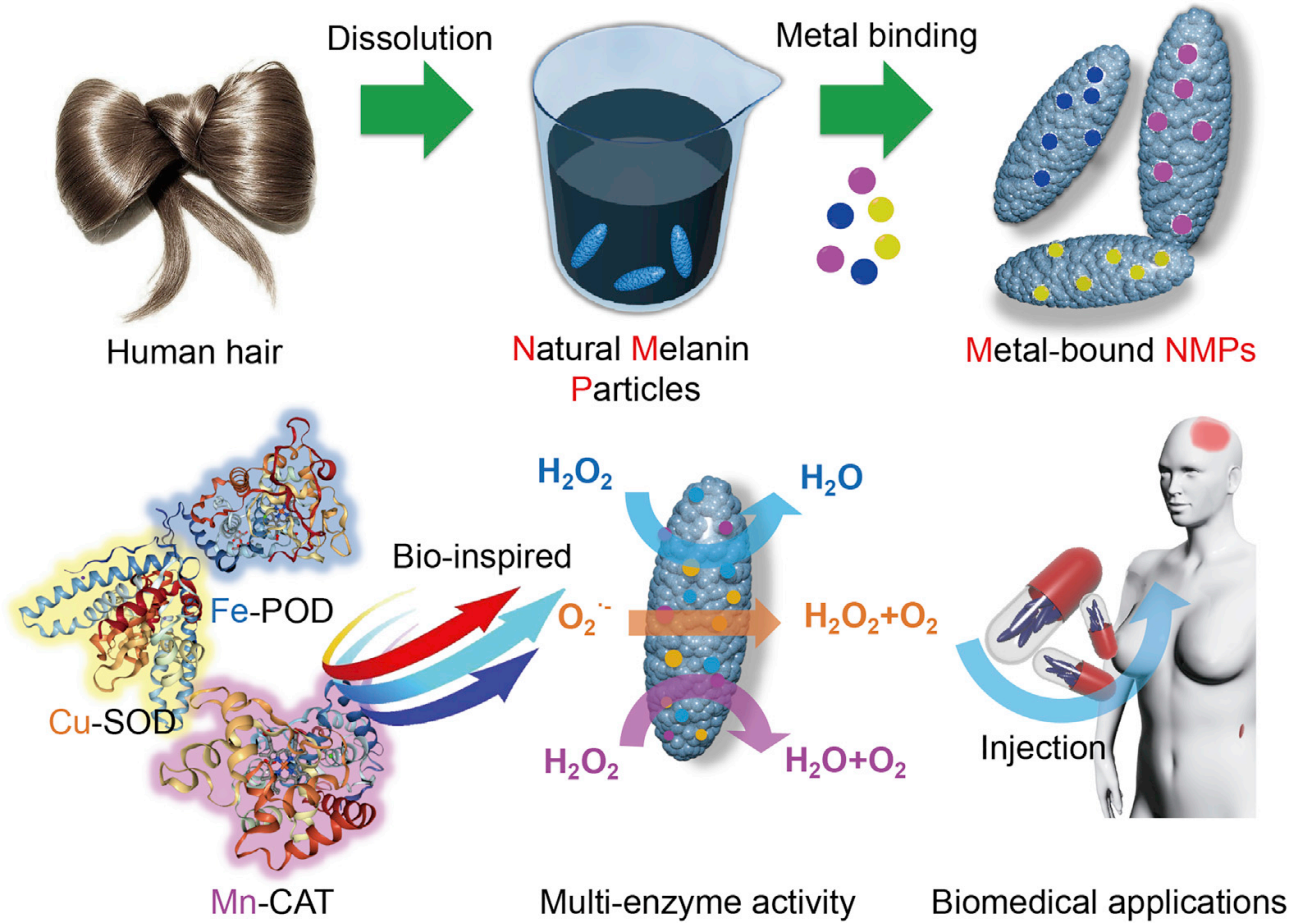
Schematic Illustration Showing the Fabrication and Multi-Enzyme Activity of Metal-Bound Natural Melanin Particles (NMPs) for Various Biomedical Applications Inspired by natural enzymes, NMPs are extracted from human hair by alkali-heat-treatment and further bound with metal ions to mimic natural enzyme activity.

It is well known that melanin in human hair exists in the form of melanin granules, which was reported to contain plenty of functional groups such as amino, carboxyl, and indolyl groups (Cho et al., 2017, Mostert et al., 2018, D’Ischia et al., 2014). In our previous study, natural melanin particles (NMPs) were extracted from human hair by using alkaline-degradation method, also confirming the presence of functional groups (Zheng et al., 2018). Here, NMPs, extracted from human hairs, were bound with metal ions to mimic the natural enzymes (Scheme 1). On the one hand, the abundant functional groups in NMPs could adsorb substrate and provide active sites for substrate binding as the protein part in natural enzymes. On the other hand, the metal ions in NMPs could also work as the metallic cofactor of enzyme (Scheme 1). It is hoped that the metal-bound NMPs (M-NMPs) derived from human hairs could serve as efficient, stable, and low-cost enzyme alternatives, providing a new view and useful tool for future biomedical applications.

## Results and Discussion

### Preparation and Characterization of M-NMPs

**Figure 1:**
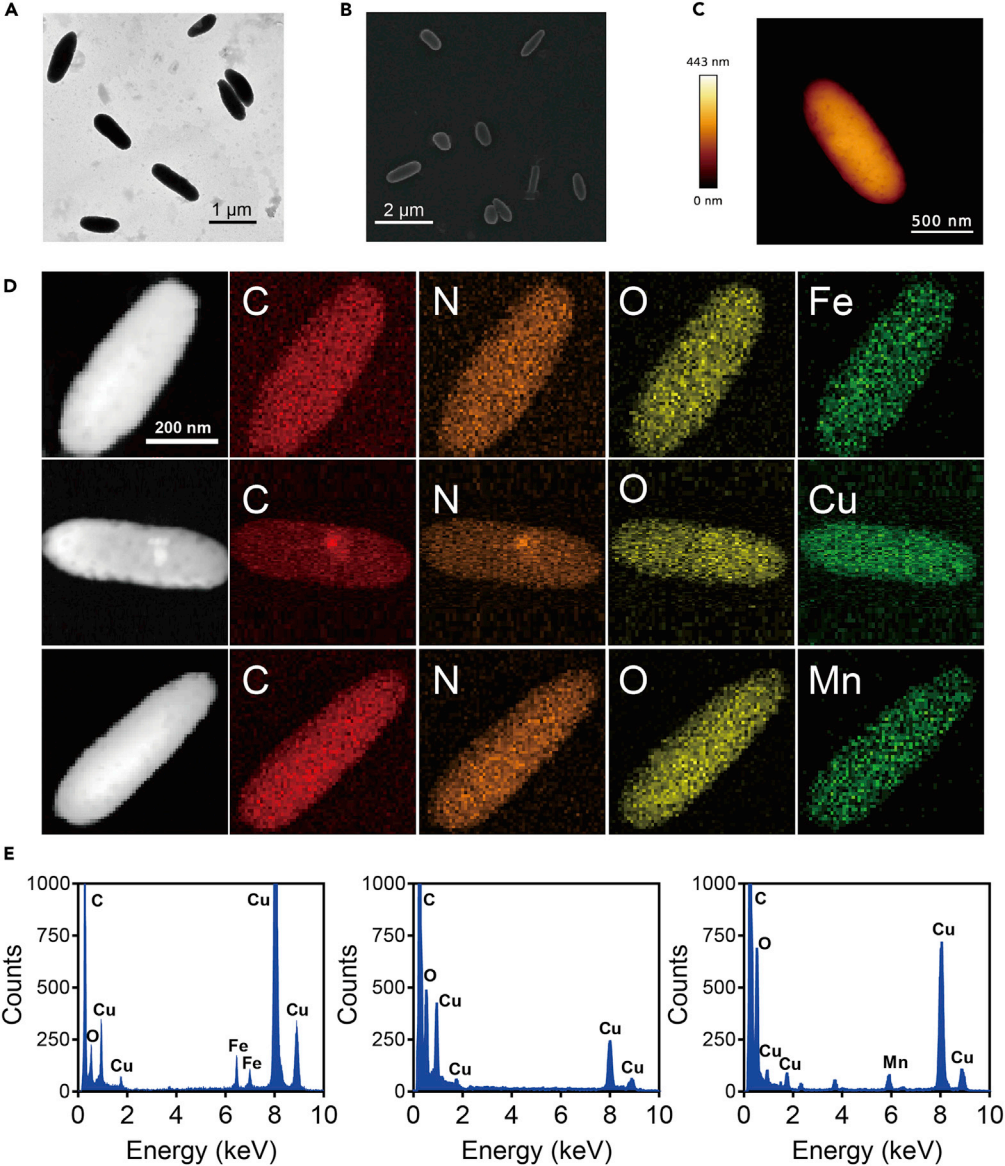
Characterization of NMPs and M-NMPs (A–C) TEM (A), SEM (B), and AFM (C) images of NMPs obtained from human hair. (D) Corresponding TEM elemental mappings of Fe-NMPs, Cu-NMPs, and Mn-NMPs. (E) Energy dispersive spectrums (EDS) of Fe-NMPs, Cu-NMPs, and Mn-NMPs. See also Figure S1 and Table S1.

The morphology and size of NMPs were characterized by transmission electron microscope (TEM), scanning electron microscope (SEM), and atomic force microscope (AFM). As presented in Figures 1A–1C, NMPs showed uniform fusiform shapes with an average length of 1.2 μm and width of 0.3 μm. Here, the Fe, Cu, and Mn ions were bound into the NMPs, and the enzyme-like activities of different M-NMPs were studied. The mapping images in Figure 1D showed a uniform distribution of C, O, N, and metal elements over the entire structure of its corresponding M-NMPs, indicating the successful binding of metal ions into NMPs. Furthermore, the energy dispersive spectrums (EDS) of M-NMPs also proved the successful binding of metal ions (Figure 1E). And the Fe, Cu, and Mn content in its corresponding M-NMPs analyzed by inductively coupled plasma mass spectrometry (ICP-MS) were 4.0wt%, 5.1wt%, and 4.2wt%, respectively (Table S1). Besides, the stability of M-NMPs was analyzed by measuring the change of mean size and the metal content, which were found to have no obvious change (Figure S1).

### Enzyme-like Activities of M-NMPs

**Figure 2:**
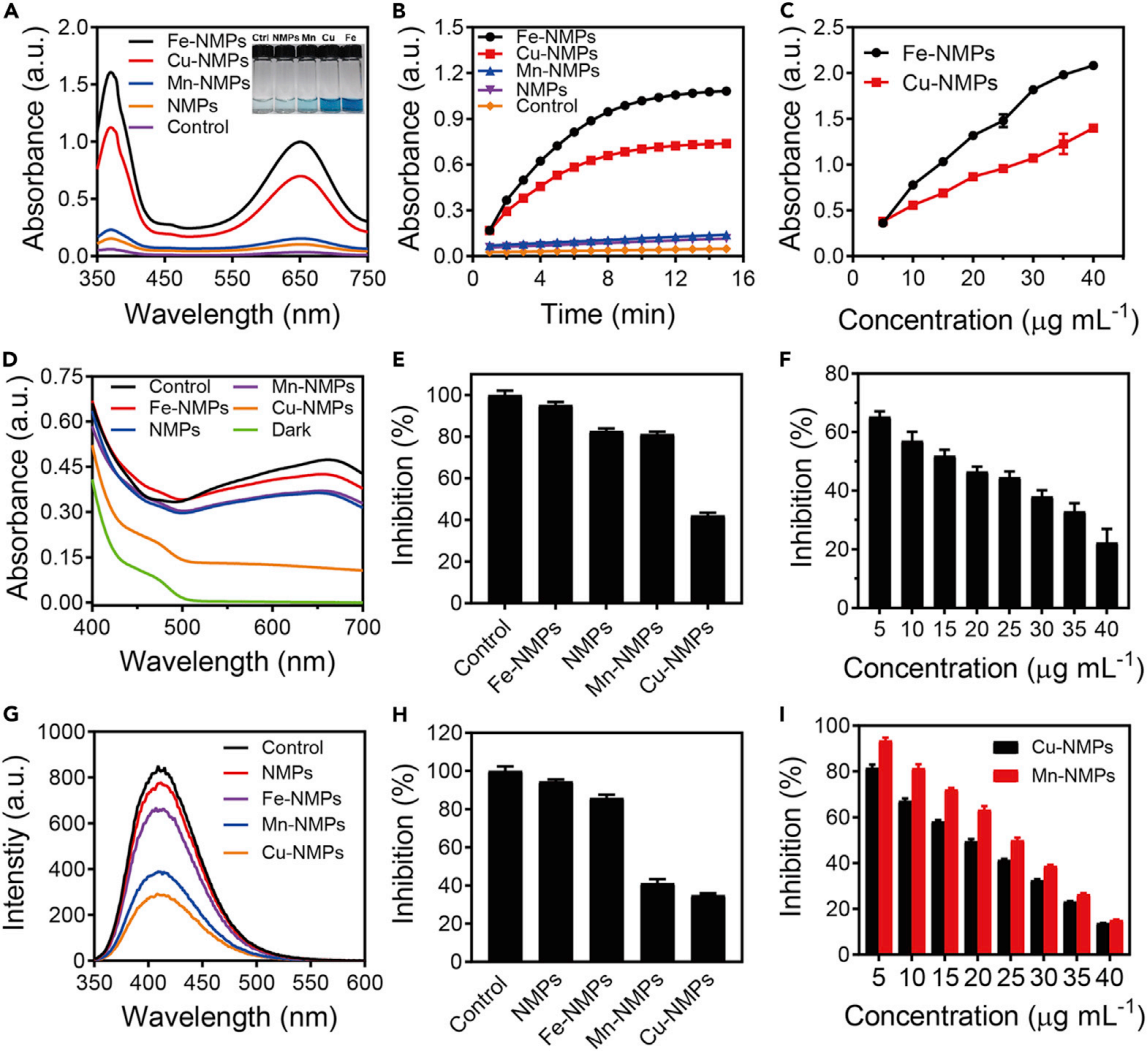
Enzyme-like Activities of M-NMPs (A) POD-like activity of different M-NMPs. Insert shows the color change of TMB oxidation with various treatments. (B) POD-like activity of different M-NMPs at different reaction time. (C) Absorbance at 650 nm of the TMB oxidation with different concentrations of Fe-NMPs and Cu-NMPs. (D) SOD-like activity of different M-NMPs. (E) Inhibition rate of NBTphotooxidation for different M-NMPs. (F) Inhibition rate of NBTphotooxidation with different concentrations of Cu-NMPs. (G) CAT-like activity of M-NMPs. (H) Inhibition rate of TA oxidation for different M-NMPs. (I) Inhibition rate of TA oxidation with different concentrations of Cu-NMPs and Mn-NMPs. Results are expressed as the mean ± S.D. of at least three independent experiments measured in triplicate. See also Figures S2–S5 and Table S2.

After successfully fabricating the M-NMPs, the enzyme-like activities were analyzed. First, the POD-like activity was evaluated by the oxidation of 3, 3′, 5, and 5′-tetramethylbenzidine (TMB). In the presence of H_2_O_2_, the colourlessTMB could be oxidized by POD to generate blue oxidized TMB (oxTMB) accompanied by an obvious increasing absorption at 650 nm (Fan et al., 2018). According to Figure 2A, the markedly increased absorption at 650 nm was observed only in the presence of Fe-NMPs and Cu-NMPs, suggesting their remarkable POD-like activity. And the color change from colourlessTMB to blue oxTMB also confirmed the same result. The effect of catalyst concentration on its activity was further investigated. It was found that the absorbance in 650 nm increased gradually with the concentration of catalysts increasing (Figures 2B, 2C, and S2). The stability of catalysts was also studied in different pH and temperature conditions. Figure S3 demonstrated that Fe-NMPs and Cu-NMPs could retain high catalytic efficiency over a wide range of pH and temperature. Here, the Michaelis-Menten equation in enzyme kinetics was introduced to intuitively assess the catalytic activity of Fe-NMPs and Cu-NMPs. The calculated Michaelis constant of the two catalysts for H_2_O_2_ and TMB indicated their comparable affinity to the substrates (Figure S4 and Table S2), demonstrating that Fe-NMPs and Cu-NMPs exhibited markedly POD-like activity.

The SOD-like activity of M-NMPs was then assayed by measuring the inhibition of the photoreduction of nitro blue tetrazolium (NBT). Under UV irradiation, the mixture of riboflavin, methionine, and NBT would produce a high level of superoxide, leading to an obvious absorption at 650 nm (Fan et al., 2018). Thus, the SOD-like activity could be evaluated by the decrease of absorption at 650 nm. As shown in Figure 2D, the mixture in the control exhibited an obvious absorption at 650 nm, whereas a sharp decrease of absorption could be observed with Cu-NMPs treatment. In the presence of 20 μg mL^−1^ of Cu-NMPs, the superoxide scavenging rates could reach to 59.8%, manifesting its excellent SOD-like activity (Figure 2E). The SOD-like activity of catalyst in different concentrations was also tested, which is found to enhance as the concentration increase (Figure 2F). These results confirmed that the Cu-NMPs showed great SOD-like activity and could be used as efficient superoxide radical removal agents.

Previous studies have demonstrated that metal ion-containing materials could catalyze the decomposition of H_2_O_2_ such as CAT (Zhang et al., 2016). Here, the CAT-like activity of M-NMPs was also investigated by measuring the inhibition of the oxidation of non-fluorescent terephthalic acid (TA). In the presence of H_2_O_2_, TA could be oxidized to generate fluorescent 2-hydroxyterephthalic acid with an emission wavelength of 425 nm (Yao et al., 2018). Therefore, the CAT-like activity of M-NMPs could be evaluated by the decrease of fluorescence intensity at 425 nm. With the treatment of Cu-NMPs and Mn-NMPs, a significant decrease of fluorescence intensity was observed, indicating that they could efficiently decompose the H_2_O_2_ (Figure 2G). And the decomposition rate could respectively reach to 65.7% and 60.4% with 30 μg mL^−1^ of Cu-NMPs and Mn-NMPs (Figure 2H). It was observed that the CAT-like activity enhanced with the increasing concentration of the catalysts (Figures 2I and S5). These results demonstrated that the Cu-NMPs and Mn-NMPs showed prominent CAT-like activity and could be used to eliminate the H_2_O_2_.

### Antibacterial Property of Fe-NMPs *In Vitro* and *In Vivo*

**Figure 3:**
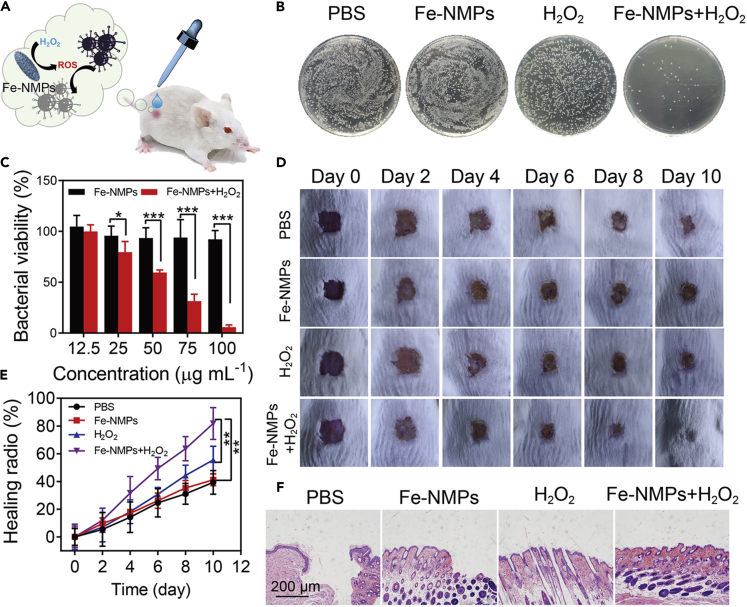
The Antibacterial Activity of Fe-NMPs against *S. aureus* (A) Schematic of the antibacterial activity of Fe-NMPs against *S. aureus*. (B) Photographs of bacterial colonies formed by *S. aureus* with different treatment. (C) Bacterial viability of *S. aureus* with different treatment. (D and E) Photographs (D) and the corresponding healing ratio (E) of *S. aureus*-infected wound with different treatment at different days. (F) H&E analysis of the wounds with different treatment after 10 days of therapy. Significance between each group was calculated using ANOVA with Tukey post hoc test. *p< 0.05, **p< 0.01, ***p< 0.001. Results are expressed as the mean ± S.D. of at least three independent experiments measured in triplicate.

Given the multienzyme-like activities of different M-NMPs, their potential applications in biomedical field were expected. Previous researches have shown that nanoparticles with POD-like property could catalyze H_2_O_2_ to form hydroxyl radicals (⋅OH) in the antibacterial applications (Xu et al., 2019). In view of the high POD-like activity of Fe-NMPs, its antibacterial property was explored (Figure 3A). Before that, the biocompatibility of the Fe-NMPs was evaluated by studying its cytotoxicity effect. According to Figure S6, Fe-NMPs exhibited good biocompatibility toward 3T3 cells, which guaranteed its applications in biomedicine. The *in vitro* antibacterial activity of Fe-NMPs toward *S. aureus* was firstly studied by using UV-vis spectrometry and plate counting method. As shown in Figure 3B, Fe-NMPs showed a good antibacterial activity toward *S. aureus* in the presence of H_2_O_2_ when compared with other groups. The same result was also observed by UV-vis spectrometry (Figure 3C). These results indicated that Fe-NMPs displayed high antibacterial activity. And the *in vivo* bactericidal efficacy of Fe-NMPs was further evaluated by using wound infection model in mice. Mice with wound infection were randomly divided into four groups and treated with PBS, Fe-NMPs (100 μg mL^−1^), H_2_O_2_ (100 μM), and Fe-NMPs + H_2_O_2_, respectively. After 10 days of treatment, the Fe-NMPs + H_2_O_2_-treated mice achieved 80.6% wound healing, whereas the controls only reached to 42.5% healing (Figures 3D and 3E). The wound healing efficiency was further verified by hematoxylin-eosin (H&E) staining (Figure 3F). Therefore, Fe-NMPs exhibited conspicuous anti-bacteria property in both *in vitro* and *in vivo* assays, revealing potential application in anti-bacterial and wound healing.

### Radiotherapy Sensitization Effect of Mn-NMPs *In Vitro* and *In Vivo*

**Figure 4:**
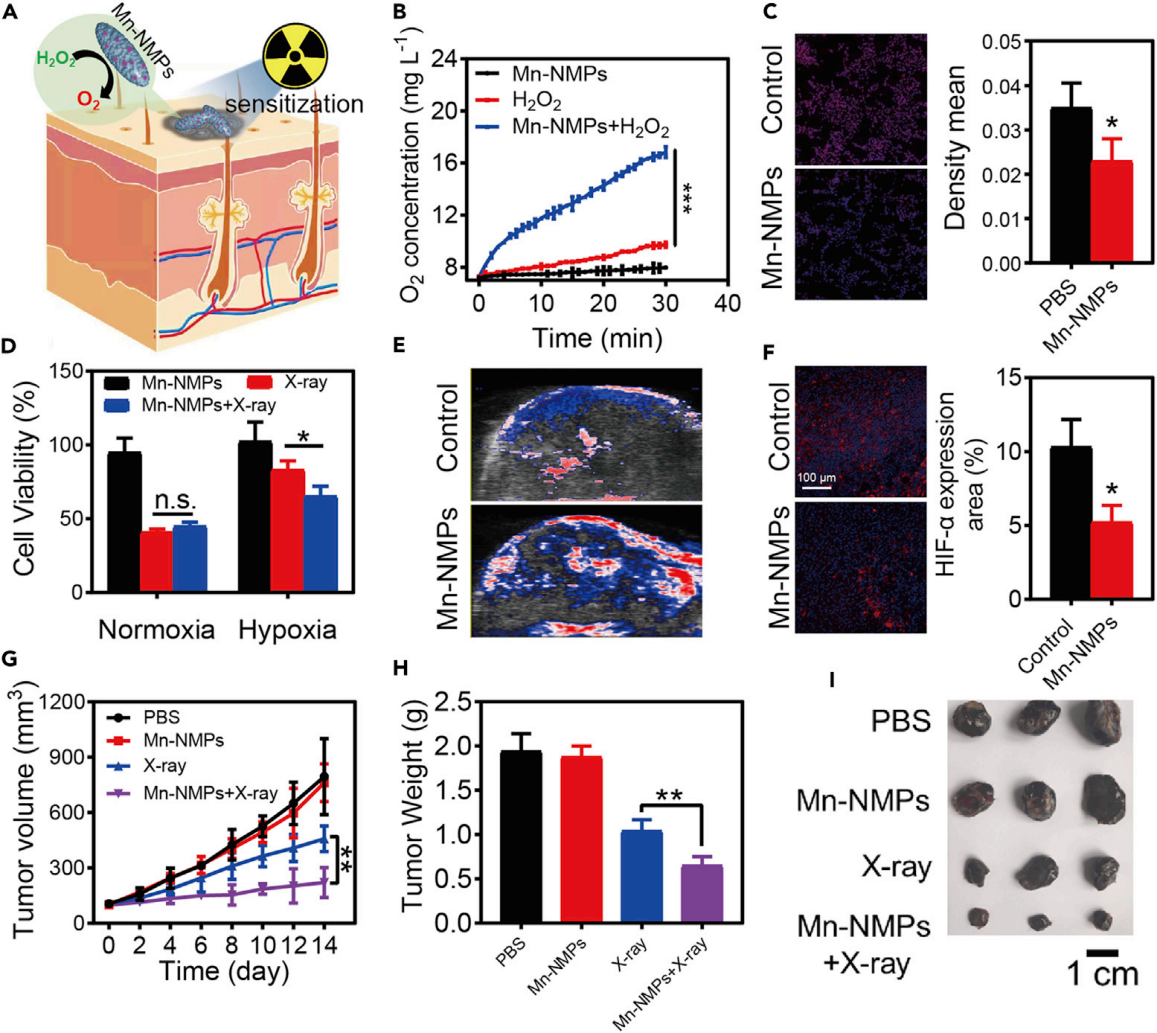
The Radiotherapy Sensitization Effect of Mn-NMPs in Cancer Therapy (A) Schematic of the radiotherapy sensitization effect of Mn-NMPs in cancer therapy. (B) Oxygen generation with different treatment. (C) HIF-1α expression level in B16-F10 cells with or without the treatment of Mn-NMPs. The cell nuclei and HIF-1α were stained with DAPI (blue) and anti-HIF-1α antibody (red), respectively. (D) Cell viability of B16-F10 cells with different treatments in normoxia or hypoxia. (E) Representative PA images of B16-F10 tumors with or without the treatment of Mn-NMPs. (F) Immunofluorescence images of B16-F10 tumor after different treatments. The cell nuclei and HIF-1α were stained with DAPI (blue) and anti-HIF-1α antibody (red), respectively. (G–I) Tumor volume changes (G), tumor weight (H), and the photograph of the tumors (I) with different treatments. Significance between each group was calculated using ANOVA with Tukey post hoc test. *p< 0.05, **p< 0.01, ***p< 0.001. Results are expressed as the mean ± S.D. of at least three independent experiments measured in triplicate. See also Figures S6–S9.

Nowadays, as one of the most common treatments for cancer in clinic, radiotherapy (RT) depends on the high-intensity ionizing radiation to activate tumor-dissolved oxygen to induced DNA damages for achieving cancer therapy (Lu et al., 2018). However, the inadequate oxygen supply in most solid tumors severely reduces the effectiveness of RT. Thus, developing methods to increase the oxygen content in the tumor tissues maybe quite a useful strategy to enhance the efficiency of RT. Catalytic decomposition of endogenous H_2_O_2_ could be an ideal strategy to increase the level of O_2_ in tumors. To date, many studies have been reported to use catalysts for endogenous H_2_O_2_ decomposition to improve the hypoxia condition of tumors for cancer treatment (Chen et al., 2019, He et al., 2019, Song et al., 2018). In consideration of the CAT-like activity of Mn-NMPs, which could act as a catalyst for decomposition of H_2_O_2_ to generate O_2_, we expected that Mn-NMPs could act as a radiosensitizer to enhance the efficiency of RT for cancer treatment (Figure 4A). Firstly, the ability of Mn-NMPs to decompose H_2_O_2_ to generate oxygen was studied by using dissolved oxygen meter. As expected, the O_2_ concentration in Mn-NMPs group was obviously increased in the presence of H_2_O_2_ (Figure 4B), confirming that the Mn-NMPs is able to decompose H_2_O_2_ to generate O_2_. Generally, the HIF-1α protein level could indicate the degree of hypoxia in cells, and the expression of HIF-1α protein would be upregulated when hypoxia. To evaluate the intracellular O_2_ generation ability of Mn-NMPs, the expression of HIF-1α protein was detected by immunofluorescence staining. Relatively low HIF-1α protein level was observed in Mn-NMPs treated cells, suggesting that Mn-NMPs could effectively alleviate the hypoxia (Figure 4C). To further investigate whether Mn-NMPs could act as radiosensitizer toward cancer cells under hypoxia, the cellular survival assay was performed in B16-F10 cells. As shown in Figure 4D, after X-ray irradiation, cells treated with or without Mn-NMPs showed a similar cellular survival rate under normoxia, whereas the Mn-NMP- treated cells showed a significant decrease in cellular survival rate under hypoxia compared with the untreated group. Notably, Mn-NMP-treated cells could effectively enhance the efficacy of RT under hypoxia, revealing that Mn-NMPs could act as a radiosensitizer for cancer treatment.

To further evaluate the radiotherapy sensitization effect of Mn-NMPs *in vivo*, a melanoma tumor model was established to study the the radiotherapy sensitization effect of Mn-NMPs. Firstly, the O_2_ generation ability of Mn-NMPs *in vivo* was studied by monitoring the saturated O_2_ levels within tumors with photoacoustic (PA) imaging. As expected, the O_2_ level was significantly increased at 6 h post-injection of Mn-NMPs (Figure 4E), indicating the ability of Mn-NMPs to decompose endogenous H_2_O_2_ into O_2_ in tumor. And the HIF-1α protein expression level was also assessed by immunofluorescence staining to confirm the O_2_ generation ability of Mn-NMPs. As exhibited in Figure 4F, the Mn-NMP-treated tumor showed relatively weak fluorescence of anti-HIF-1α antibody compared with the control, indicating the downregulation of HIF-1α expression through O_2_ generation. These results indicated that the Mn-NMPs could relieve the tumor hypoxia through the decomposition tumor endogenous H_2_O_2_ into O_2_. Next, the *in vivo* antitumor ability of Mn-NMPs was evaluated to study its radiotherapy sensitization effect. According to the tumor volume and the tumor weight evaluated on the 14th day, Mn-NMP-treated group showed unconspicuous therapeutic effect without X-ray irradiation. A slight tumor inhibition was observed with X-ray irradiation alone, whereas Mn-NMP-treated group showed an obvious inhibition of tumor growth with 92.6% tumor suppression under X-ray irradiation, suggesting superior radiotherapy sensitization effect of Mn-NMPs in hypoxia (Figures 4G and 4H). And the images of tumor size and the histologic section assays also confirmed the positive therapeutic effect of the radiosensitizer (Figures 4I and S7). No obvious body weight changes were observed during the treatment, and no abnormality was found in major organs after the treatment, illustrating little systemic toxicity of Mn-NMPs (Figures S8 and S9). These results suggested that Mn-NMPs could provide oxygen through decomposing endogenous H_2_O_2_ in tumors and act as a radiosensitizer to enhance RT efficacy.

### Anti-inflammatory Effect of Cu-NMPs *In Vitro* and *In Vivo*

**Figure 5:**
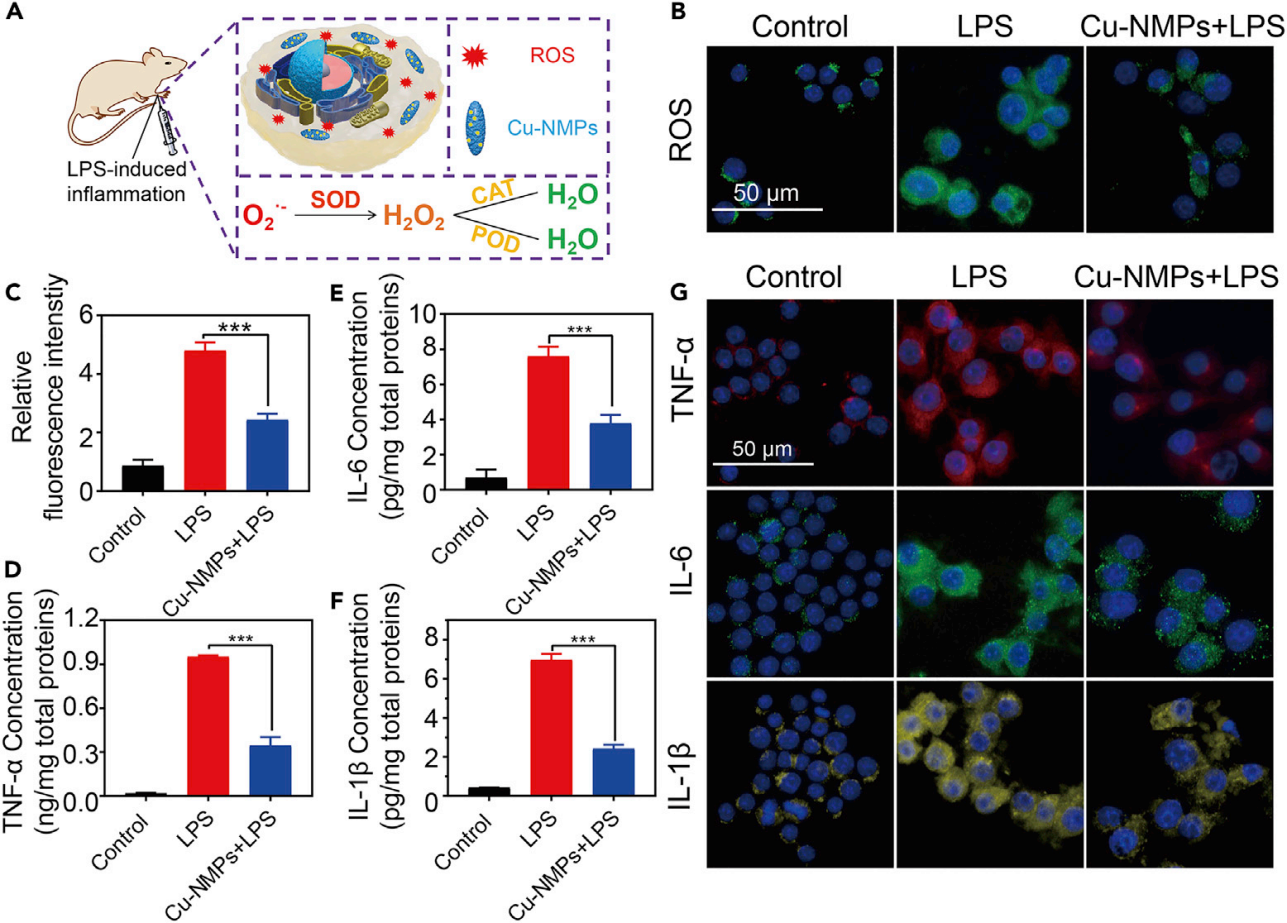
The Anti-inflammation Effect of Cu-NMPs toward RAW264.7Cells (A) Schematic of the anti-inflammation effect of Cu-NMPs toward RAW264.7 cells. (B) Fluorescence microscopy images of ROS level in RAW264.7 cells with different treatments. (C) ROS level in RAW264.7 cells with different treatments.| (D–F) The expression level of pro-inflammatory cytokines TNF-α (D), IL-6 (E), and IL-1β (F) in RAW264.7 cells with different treatments. (G) Fluorescence microscopy images of pro-inflammatory cytokines TNF-α, IL-6, and IL-1β expression in RAW264.7 cells with different treatments. Significance between each group was calculated using ANOVA with Tukey post hoc test. ***p< 0.001, n.s. = not significant. Results are expressed as the mean ± S.D. of at least three independent experiments measured in triplicate. See also Figures S10–S15.

In human body, harmful superoxide radicals are transformed into H_2_O_2_ through SOD. And then the H_2_O_2_ is further decomposed into completely harmless water by CAT and POD. In this way, the three enzymes form a complete anti-oxidation chain to protect cells against oxidative damage (Huang et al., 2016). Given that the Cu-NMPs were verified to show the three enzyme-like activities of anti-oxidation chain simultaneously, we expected that the Cu-NMPs could rescue the oxidative stress and show the potential of remedying oxidation-related diseases. Inflammation, a disease linked to ROS-induced oxidative stress, could be alleviated by eliminating the harmful ROS (Zhang et al., 2019, Wan et al., 2017). So the anti-inflammation ability of Cu-NMPs was studied to assess its therapeutic potential in oxidation-related diseases (Figure 5A). Before that, the biocompatibility of the Cu-NMPs was evaluated by studying its cytotoxicity effect toward mouse macrophage cell line (RAW264.7). After incubating RAW264.7 with Cu-NMPs at different concentrations for 24 h, it was observed that the cell proliferation was hardly affected (Figure S10). This result demonstrated that Cu-NMPs possessed favorable biocompatibility and could be applied in biomedical applications. LPS, which exists as antigen in the outer membrane of bacteria, has been used as a pattern in the induction of inflammation models. Here, the anti-inflammatory action of Cu-NMPs was examined on the LPS-induced inflammation of RAW264.7. Having been stimulated by LPS, the macrophages would produce an inflammatory reaction resulting in the excess production of ROS and the high expression level of pro-inflammatory cytokines such as TNF-α, IL-6, and IL-1β. The ROS level was analyzed by detecting the conversion of non-fluorescent 2′, 7′-dichlorofluoresceindiacetate (DCFH-DA) to strong fluorescent 2′, 7′-dichlorofluorescein (DCF) (Zhang et al., 2015). According to the fluorescence microscopy images in Figures 5B and S11, intense green fluorescence was observed in LPS-incubated RAW264.7. In contrast, after being treated with Cu-NMPs, the macrophages showed a dramatical decreased fluorescence, indicating the ROS scavenging capacity of the Cu-NMPs (Figure 5C). To further assess the anti-inflammation ability of Cu-NMPs, the expression of three pro-inflammatory cytokines, TNF-α, IL-6, and IL-1β, in RAW264.7 were quantified by enzyme-linked immunosorbent assay (ELISA). As shown in Figures 5D–5F, LPS treatment led to an overexpression of three pro-inflammatory cytokines in macrophages, which significantly decreased under the treatment of Cu-NMPs. With 40 μg mL^−1^ of Cu-NMPs treatment, the removal rate of TNF-α, IL-6, and IL-1β could reach to 65.1%, 55.5%, and 69.4%, respectively (Figure S12). Immunocytochemistry staining was also employed to measure the expression level of TNF-α, IL-6, and IL-1β. It was observed that LPS-stimulated overexpression of pro-inflammatory cytokines in macrophages were drastically reduced after the treatment of Cu-NMPs (Figures 5G and S13–S15). These *in vitro* results demonstrated that Cu-NMPs could effectively reduce inflammatory responses and so may have the potential to be applied in oxidation-related diseases.

**Figure 6:**
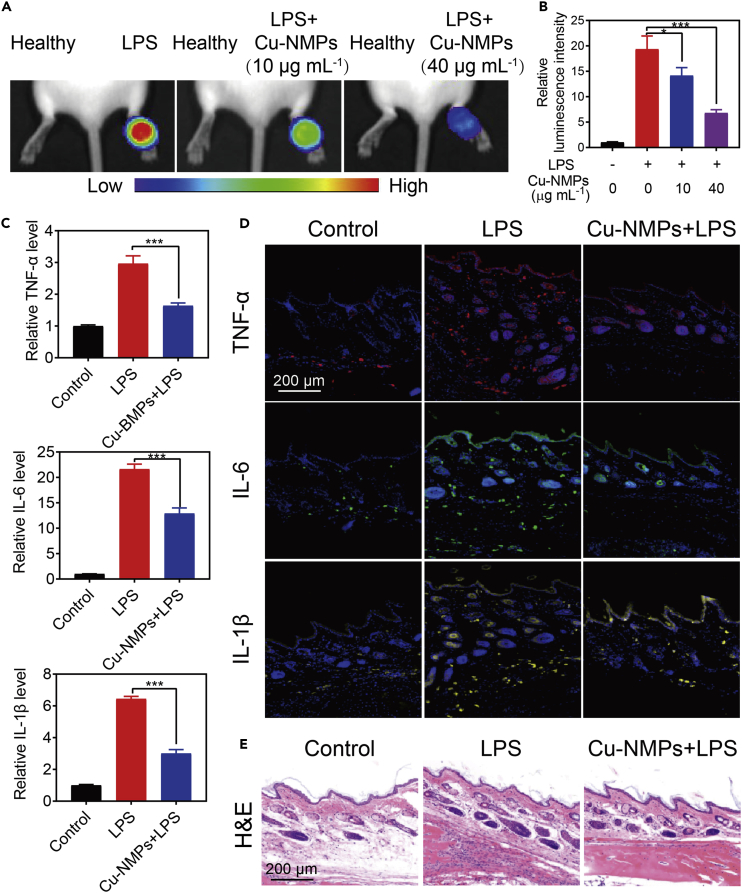
The Anti-inflammation Effect of Cu-NMPs on LPS-Induced Inflamed Paws (A and B) *In vivo* bioluminescence imaging (A) and corresponding luminescence intensities (B) of ROS level in LPS-induced inflamed paws with different treatments. (C) The expression level of pro-inflammatory cytokines TNF-α, IL-6, and IL-1β in LPS-induced inflamed paws with different treatments. (D) Immunofluorescence staining of pro-inflammatory cytokines TNF-α, IL-6, and IL-1β expression in LPS-induced inflamed paws with different treatments. (E) H&E staining images of LPS-induced inflamed paws of mice with different treatments. Significance between each group was calculated using ANOVA with Tukey post hoc test. *p< 0.05, ***p< 0.001. Results are expressed as the mean ± S.D. of at least three independent experiments measured in triplicate. See also Figures S16–S20.

We also established an inflammation model on BALB/c mice to further explore the potential application of Cu-NMPs in anti-inflammation *in vivo*. The inflammation model on paw of BALB/c mice was constructed by local injection of LPS. The ROS level in paws was imaged by a luminescent probe. As displayed in Figures 6A and 6B, strong luminescence signal of ROS was detected in LPS-induced inflamed paws, whereas the ROS level obviously decreased in the presence of Cu-NMPs. It was found that the luminescence signal decreased progressively with the increasing concentrations of Cu-NMPs. Cu-NMPs showed 70.0% ROS removal rate at 40 μg mL^−1^, revealing an admirable ROS scavenging activity (Figure S16). To further evaluate the anti-inflammation ability of Cu-NMPs*in vivo*, the expression of TNF-α, IL-6, and IL-1β in LPS-induced inflammatory paw was quantified by ELISA. It was found that LPS-induced overexpression of pro-inflammatory cytokines were dramatically decreased with the treatment of Cu-NMPs (Figure 6C). A dose-dependent decrease in the expression of pro-inflammatory cytokines was also observed in mice treated with Cu-NMPs. The removal rate of the TNF-α, IL-6, and IL-1β could respectively reach to 96.7%, 67.2%, and 83.5% with 40 μg mL^−1^ of Cu-NMPs (Figure S17). Immunofluorescence staining was also used to visualize the expression of TNF-α, IL-6, and IL-1β in inflamed paws. Treatment with Cu-NMPs significantly reduced the LPS-induced pro-inflammatory cytokines levels, demonstrating the effectiveness of Cu-NMPs in inhibiting the production of inflammatory cytokines in tissue inflammation (Figures 6D and S18–S20). H&E staining was also assayed to measure the infiltration of the inflammatory cells. A markedly enhanced infiltration of the inflammatory cells was observed in inflamed paws, which was significantly decreased after the treatment of Cu-NMPs (Figure 6E). These *in vivo* results demonstrated that Cu-NMPs could effectively reduce inflammatory responses, showing the possibility of applying in oxidation-related diseases.

### Limitations of the Study

M-NMPs based on the human hairs succeeded in mimicking natural enzymes and exhibited some catalytic activities. However, what cannot be ignored is the variability of hair samples. In this study, the hair samples we used were mainly the hairs of yellow race obtained from nearby barbershop. The hairs in different racial types have different constitutions and constructions (Imai et al., 2016, Liu et al., 2005), which may possess different activity from our enzyme-like system. Owing to the lack of the hair samples from different racial types, the activity of enzyme-like system derived from other hair samples were not studied. Furthermore, the refined structure and catalytic mechanism of M-NMPs need to be further explored for better understanding the catalytic mechanism. Besides, as an enzyme-like system, the selectivity of the mimic enzyme appears to be particularly important and the ability of selective catalysis for this enzyme-like system should be improved.

In summary, a novel natural enzyme alternative was prepared by binding different metal ions into NMPs extracted from human hair. The different M-NMPs exhibited different enzyme-like activities. The Fe-NMPs and Mn-NMPs showed outstanding POD-like activity and CAT-like activity respectively. In addition, the Cu-NMPs could serve as combined POD, SOD, and CAT alternatives to mimic anti-oxidation chain to eliminate ROS. The enzyme-active M-NMPs have the potential to be applied in the biomedical application. Both *in vitro* and *in vivo* antibacterial activity assays demonstrated that Fe-NMPs possessed the potential in antibacterial applications. Besides, Mn-NMPs with CAT-like activity exhibited a remarkable radiotherapy sensitization effect in cancer therapy. Furthermore, Cu-NMPs showed three enzyme-like activities of anti-oxidation chain simultaneously and could rescue the oxidative stress to protect cells against oxidative damage with the potential of remedying oxidation-related diseases. Anyway, this study might provide new perspectives on developing enzyme alternatives with multifunctionality to meet various requirements in biomedical applications.

## Methods

All methods can be found in the accompanying Transparent Methods supplemental file.

## Supplemental Information

Document S1. Transparent Methods, Figures S1–S20, and Tables S1 and S2
