# Supplementary material for: Enzyme Mimicking Based on the Natural Melanin Particles from Human Hair
Source: iScience. 2019 Dec 18;23(1):100778. doi: 10.1016/j.isci.2019.100778 (PMC6948237; doi:10.1016/j.isci.2019.100778)
Supplement: Document S1. Transparent Methods, Figures S1–S20, and Tables S1 and S2 [file mmc1.pdf]

ISCI, Volume *23*

Supplemental Information

Enzyme Mimicking Based on the Natural Melanin Particles from Human Hair

Sheng Hong, Qiu-Ling Zhang, Di-Wei Zheng, Cheng Zhang, Yu Zhang, Jing-Jie Ye, Han Cheng, and Xian-Zheng Zhang

Transparent Methods

Materials

Hydrogen peroxide (H_2O_2) were obtained from Sigma. Riboflavin was purchased from Sinopharm Chemical Reagent Co., Ltd. Methionine, glutathione (GSH), 3,5,3',5'-tetramethylbenzidine (TMB) and terephthalic acid (TA) were purchased from Aladdin Industrial Corporation. Nitrotetrazolium blue chloride (NBT) were purchased from Meryer (Shanghai) Chemical Technology Co., Ltd. Hoechst 33342 and DAPI were purchased from Thermofisher Scientific. 3-(4,5-dimethyl-2-thiazolyl)-2,5-diphenyl-2-H-tetrazolium bromide (MTT) assay were purchased from Beyotime Biotechnology. The hairs were obtained from the barber shop nearby.

Preparation of NMPs and M-NMPs

The NMPs were prepared according to our previous work. Briefly, 5 g human hair was dissolved in 50 mL NaOH solution (1 M) and heated to 85°C for 5 min. Then, the dark coloured solution was dialyzed against PBS for several times. Next, the NMPs were obtained by differential centrifugation with 200 g for 6 min to remove large residues and 1200 g for 10 min to gather the products. Finally, the NMPs were washed with DI water for several times and dried for later use.

The M-NMPs were prepared as follows. The prepared NMPs were stirred in 100 mM NaC1 solutions contained 10 mM corresponding metal ions at room temperature for 12 h. The metal-bound NMPs were obtained by centrifugation and dried for later use.

POD-like activity of M-NMPs

The POD-like activity of M-NMPs was performed in HAc-NaAc buffer solution (pH 3.6) by studying the oxidation of TMB with H_2O_2. Typically, the oxidation of TMB

was carried out in a mixture of M-NMPs solution (1 mg mL^{-1}, 20 μL), H_2O_2 (30%, 120 μL), TMB (10 mg mL^{-1}, 40 μL) in HAc-NaAc buffer solution with final volume of 1 mL. The absorbance of the mixture at 650 nm was recorded continuously at different reaction time. The UV-Vis absorption spectrum of the mixture was measured at 10 min of the reaction. And the change in color was photographed in the end of the reaction. The concentration dependence of catalysis was studied gradually from 5 μg mL^{-1} to 40 μg mL^{-1} of M-NMPs. The POD-like catalytic stability was assayed at different pH (2-8) and temperature (20-70 °C) conditions. The absorbance of the mixture at 650 nm with different pH and temperature was recorded.

The steady-state kinetic assays were conducted in 200 μL buffer solution with Fe-NMPs and Cu-NMPs as catalyst in the presence of different concentrations of H_2O_2 and TMB. The kinetic assays with TMB as the substrate were performed by the mixture of catalyst (10 μg mL^{-1}), 30% H_2O_2 (24 μL) and different concentrations of TMB solution (41.6, 83.2, 166.4, 249.6, 332.8, 416.0, 520.0, 624.0, 728.0, 832.0 μM). And the kinetic assays with H_2O_2 as the substrate were performed by the mixture of catalyst (10 μg mL^{-1}), TMB (10 mg mL^{-1}, 10 μL) and different concentrations of H_2O_2 (0.0441, 0.0882, 0.1764, 0.2646, 0.3528, 0.441, 0.5292, 0.6174, 0.7056, 0.882, 1.323, 1.764 M). The absorbance of the reactions at 650 nm was recorded continuously at different reaction time. And the Michaelis-Menten constant was calculated according to the Michaelis-Menten saturation curve fitting by GraphPad Prism 7.

SOD-like activity of M-NMPs

The SOD-like activity of M-NMPs was tested by measuring the inhibition of the photoreduction of NBT. In brief, the M-NMPs mixed with riboflavin (20 μM), methionine (13 mM), NBT (75 μM) in PBS (25 mM, pH 7.4) were illuminated by UV light for 10 min. After that, the UV-Vis absorption spectrum of the mixture was

measured. The mixture treated without M-NMPs and kept in the dark were served as the control and the blank, respectively. The inhibition rate was calculated by the equation: inhibition rate (%) = $[(A_0-A)/A_0] \times 100$ (A_0 and A refer to the absorbance of the control and the sample, respectively).

CAT-like activity of M-NMPs

The CAT-like activity of Cu-NMPs was analyzed by measuring inhibition of the generation of highly fluorescent 2-hydroxyterephthalic acid from non-fluorescent TA. In the presence of H_2O_2, TA could be oxidized to generate fluorescent 2-hydroxyterephthalic acid with a fluorescence signal at 425 nm upon excitation wavelength of 320 nm. The mixture containing M-NMPs, TA (0.5 mM) and H_2O_2 (10 mM) in PBS (25 mM, pH 7.4) were incubated for 24 h at room temperature. The fluorescence spectrum of mixture was then measured with an excitation wavelength of 320 nm.

Bacterial culture

Staphylococcus aureus (ATCC 25923) were cultured in Luria-Bertani (LB) medium at 37 °C. The LB medium contained 10 mg mL^{-1} tryptone, 5 mg mL^{-1} yeast extract and 0.5 mg mL^{-1} NaCl.

***In vitro* antibacterial experiments**

Firstly, UV–vis spectroscopy was performed to evaluate the antibacterial property of Fe-NMPs against *S. aureus*. Briefly, the *S. aureus* suspension were added into each well of a 96 well plate and separately treated with PBS, different concentration of Fe-NMPs and Fe-NMPs + H_2O_2. After incubation at 37 °C for 8 h, the absorbance of the suspension at 600 nm was measured to assess the bacterial viability.

The antibacterial effect of Fe-NMPs was also studied with plate counting method. *S. aureus* in different four groups were treated with PBS, Fe-NMPs (50 μg mL^{-1}),

H_2O_2 (100 μM) and Fe-NMPs + H_2O_2, respectively. The mixtures were then reacted for 30 min followed by placing on the LB solid medium and incubated for another 24 h. Counting the number of colonies in each group. All experiments were repeated three times.

Cell culture

RAW264.7 cells were cultured in 1640 medium with 5% CO_2 at 37 °C. B16-F10 cells were cultured in DMEM medium with 5% CO_2 at 37 °C. The 1640 medium and DMEM medium contained 10% heat-inactivated FBS and 1% antibiotics (penicillin-streptomycin, 10000 U mL^{-1}).

***In vitro* cell viability**

The *in vitro* cytotoxicity of Mn-NMPs against B16-F10 cells was detected by MTT assay. The B16-F10 cells were seeded in 96 well plates and followed by incubated with Fe-NMPs. 24 h later, the cells were treated with X-ray radiation (4 Gy). After 24 h post-irradiation, 20 μL of MTT (5 mg mL^{-1}) was added into each well and incubated for another 4 h. Subsequently, the culture medium containing MTT was replaced with DMSO (150 μL and the absorbance at 570 nm was determined using a microplate reader. The relative cell viability was calculated.

The *in vitro* cytotoxicity of Cu-NMPs towards RAW264.7 cells was also detected by MTT assay. RAW264.7 cells were seeded in 96-well plates (5×10^4 cells per well). 24 h later, the cells were incubated with Cu-NMPs at various concentrations (0, 0.625, 1.25, 2.5, 5, 10, 20, 40 μg mL^{-1}). Following incubation for another 24 h, the cell viability was evaluated using a MTT assay.

***In vitro* cytokines production**

The anti-inflammation ability of Cu-NMPs was studied by the measuring the expression level of pro-inflammatory cytokines TNF-α, IL-6 and IL-1β in

LPS-stimulated RAW264.7 cells. The RAW264.7 cells were seeded in 6-well plates. After 24 h of growth, the cells were treated with LPS for 6 h for inducing a inflammatory reaction. Afterwards, the LPS-induced cells were treated with Cu-NMPs. The supernatant was then collected and analyzed by ELISA to quantify the levels of TNF-α, IL-6 and IL-1β in each sample. And the expression levels of three pro-inflammatory cytokines were also visualized using immunocytochemical staining. After being washed with PBS for several times, the cells were fixed with 4% formaldehyde. The fixed cells were then stained with the antibodies for the three cytokines and DAPI. The images were observed by using CLSM.

Animal

Experimental protocols were approved by the Institutional Animal Care and Use Committee (IACUC) of the Animal Experiment Center of Wuhan University (Wuhan, China). All animal experimental procedures were performed in accordance with the Regulations for the Administration of Affairs Concerning Experimental Animals approved by the State Council of People's Republic of China.

***In vivo* wound model and antibacterial effect**

The antibacterial effect of Fe-NMPs was performed by wound infection model on female Balb/c mice. A wound of d = 10 mm was created on the back of the mice by surgery followed by injection of 10^8 CFU *S. aureus* to the wounds to build the wound infection model. The mice with infected wounds were divided into four groups (five mice per group) and treated with PBS, Fe-NMPs (100 μg mL^{-1}), H_2O_2 (100 μM) and Fe-NMPs + H_2O_2 through subcutaneous injection, respectively. The wounds were photographed every two days. After 10 days of treatments, the mice were sacrificed and the wound tissues were harvested and analyzed by H&E staining.

***In vivo* antitumoral effect**

The B16-F10 melanoma model was used as an example to evaluate the *in vivo* antitumoral effect. B16-F10 cells (1×10^6) in 100 μL of PBS were injected subcutaneously into the back of C57 male mice. When the size of tumor reached ~ 100 mm^3, the mice were randomly divided into four groups (five mice per group) and treated with PBS, Mn-NMPs, X-ray and Mn-NMPs+X-ray through subcutaneous injection, respectively. The X-ray irradiation (8 Gy) was carried out after Mn-NMPs injection for 12 h. The tumor sizes and body weights were measured every day for 14 days post-treatment.

***In vivo* inflammation models and anti-inflammatory effect**

The inflammation models on paw of BALB/c mice were constructed by local injection of LPS (20 μL, 2 mg mL^{-1}) in the paws of mice. After 6 h stimulation, the paws were treated with Cu-NMPs through subcutaneous injection (five mice per group). The levels of ROS in the inflamed paws were imaged by bioluminescence imaging on an IVIS imaging system. And the expression levels of pro-inflammatory cytokines TNF-α, IL-6 and IL-1β in the inflamed paws were also analyzed by ELISA.

Statistical analysis and sample collection

Significance among more than two groups was calculated using ANOVA Turkey's test by using SPSS 22.0. For cell experiments and *in vivo* experiments, investigators performing operations were blinded to treatment groups. In *in vivo* experiments, animals were randomly divided into different groups.

Supplemental Figures

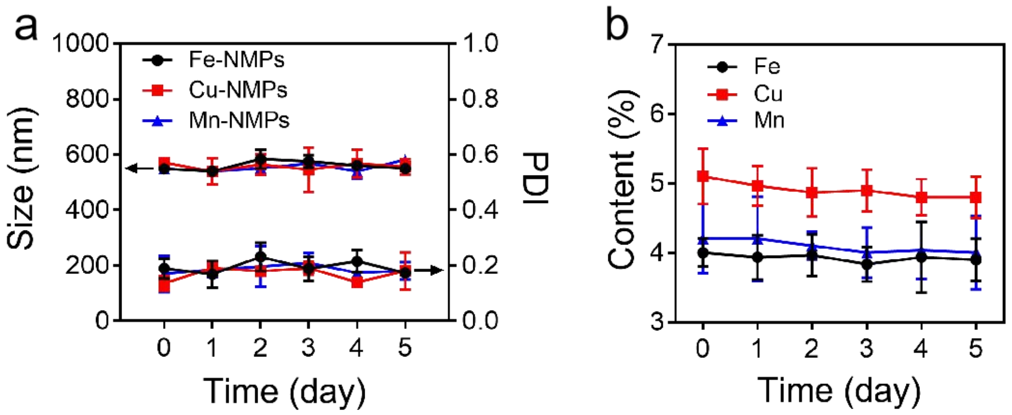

Figure S1. Related to **Figure 1.** The stability of M-NMPs. (a) Mean size and PDI of M-NMPs in 5 days. (b) Metal content of M-NMPs in 5 days.

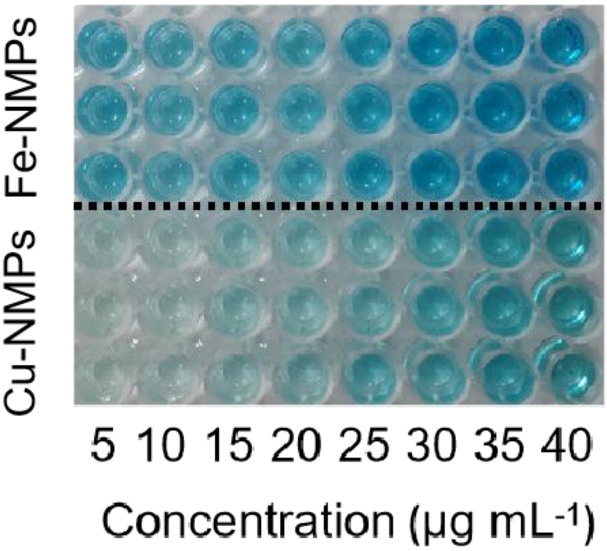

Figure S2. Related to **Figure 2.** The color changes of the TMB oxidation with different concentrations of Fe-NMPs and Cu-NMPs.

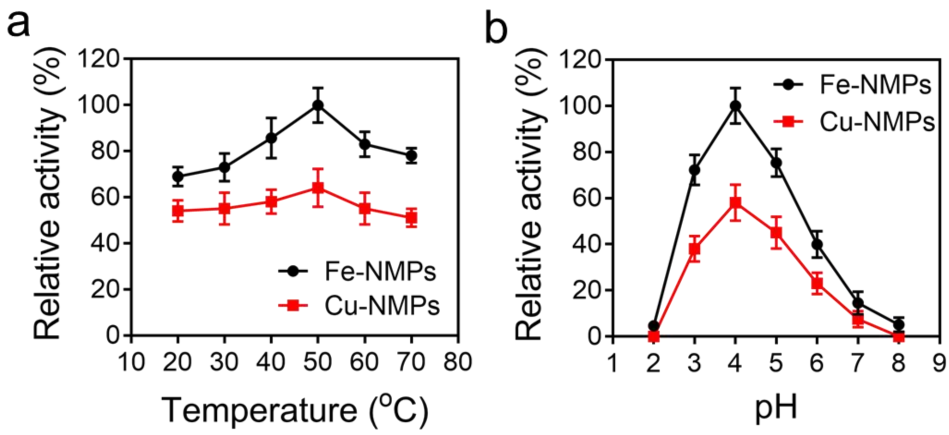

Figure S3. Related to **Figure 2.** Characterization for POD-like activity. The relative POD-like activity of Fe-NMPs and Cu-NMPs at different temperature (a) and different pH (b). Results are expressed as the mean ± S.D. of at least three independent experiments measured in triplicate.

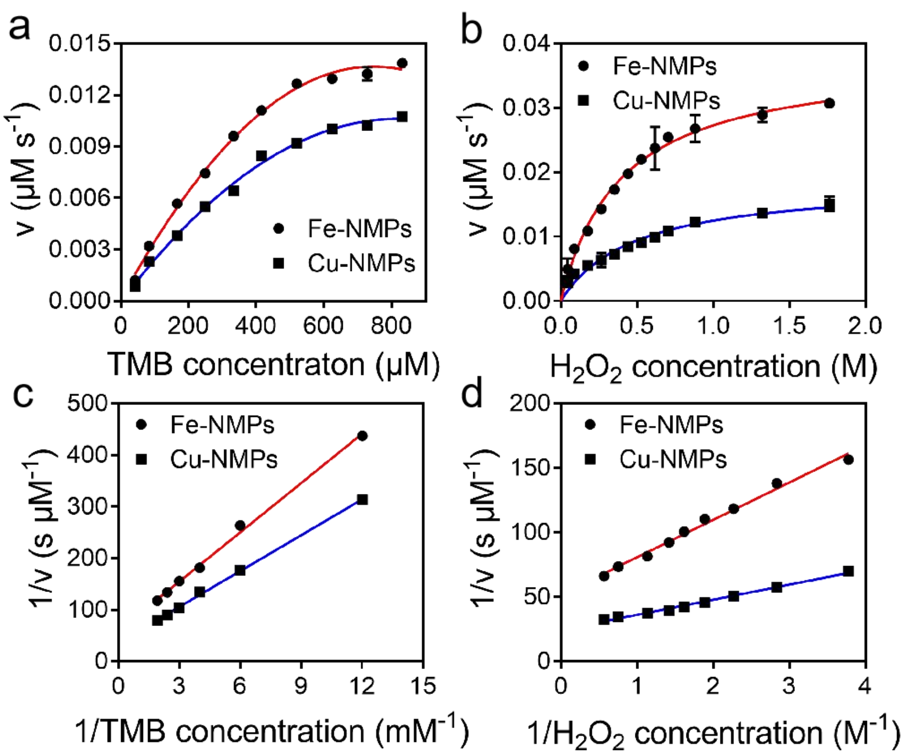

Figure S4. Related to **Figure 2.** The steady-state kinetic assays of Fe-NMPs and Cu-NMPs as catalysts and different concentrations of H_2O_2 and TMB as substrates.

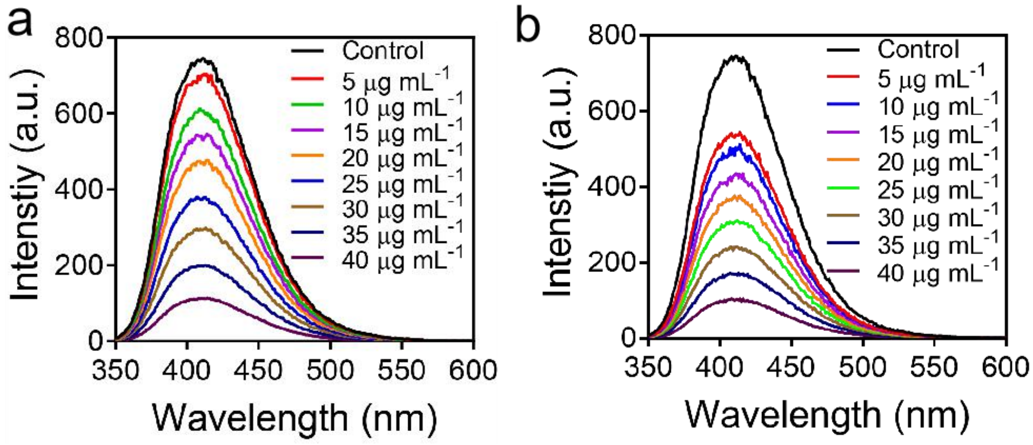

Figure S5. Related to **Figure 2.** The decomposition efficiency of H_2O_2 in presence of different concentrations of Mn-NMPs (a) and Cu-NMPs (b).

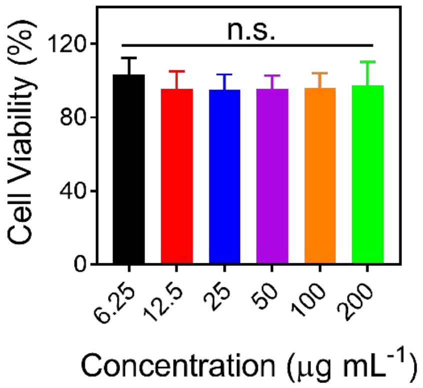

Figure S6. Related to **Figure 4.** Cell viability of 3T3 cells with the treatment of different concentration of Fe-NMPs.

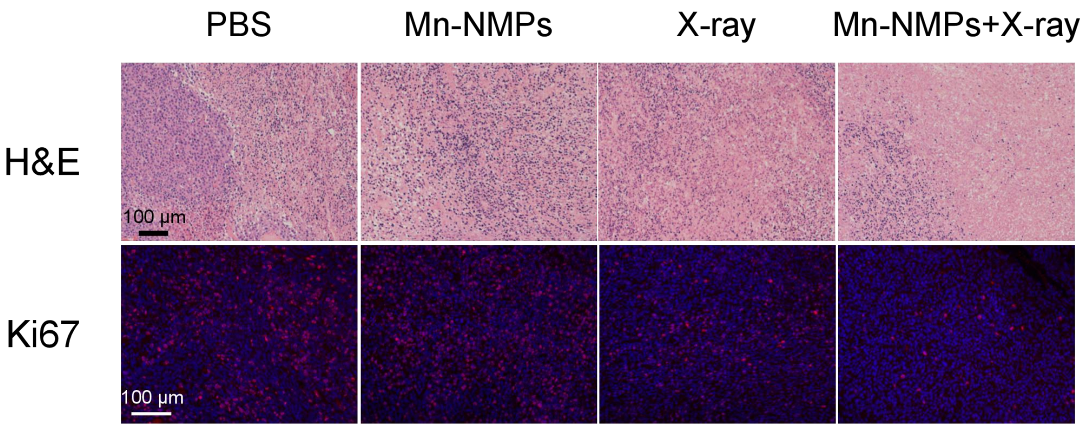

Figure S7. Related to **Figure 4.** H&E and Ki67 immunofluorescence staining of B16-F10 tumor tissues with different treatment.

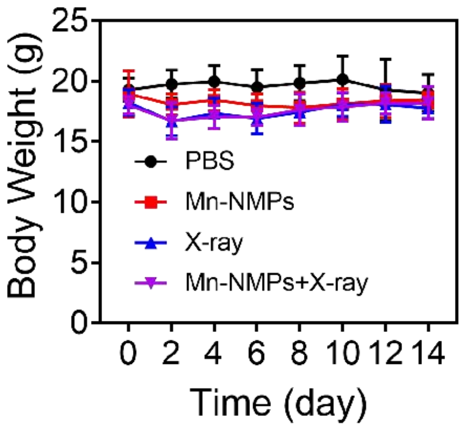

Figure S8. Related to **Figure 4.** Body weight changes of mice with different treatment.

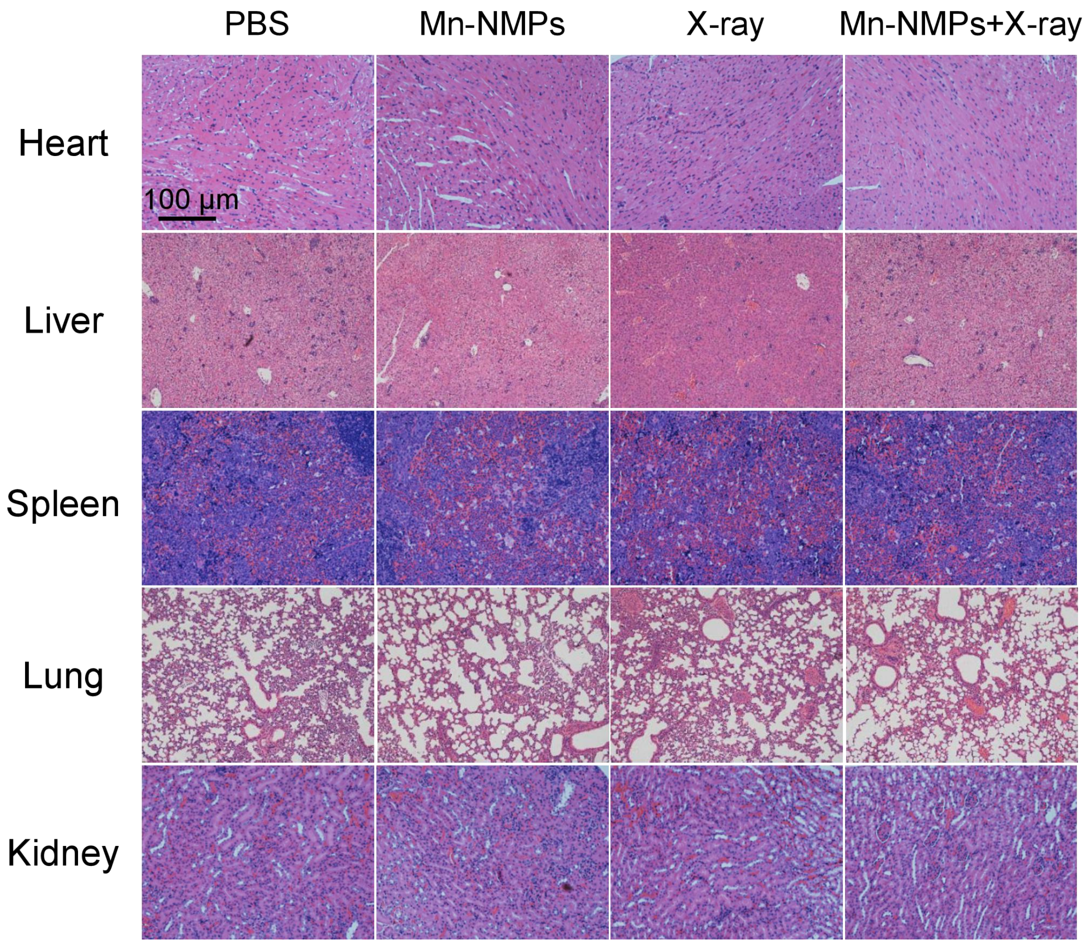

Figure S9. Related to **Figure 4.** H&E staining of mice hearts, livers, spleens, lungs and kidneys with different treatment.

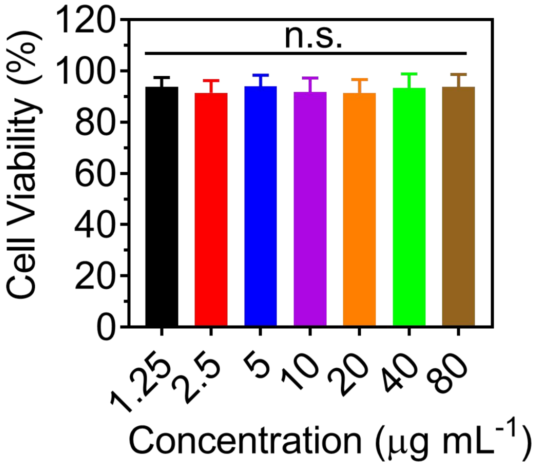

Figure S10. Related to **Figure 5.** Cell viability of RAW246.7 with the treatment of different concentration of Cu-NMPs.

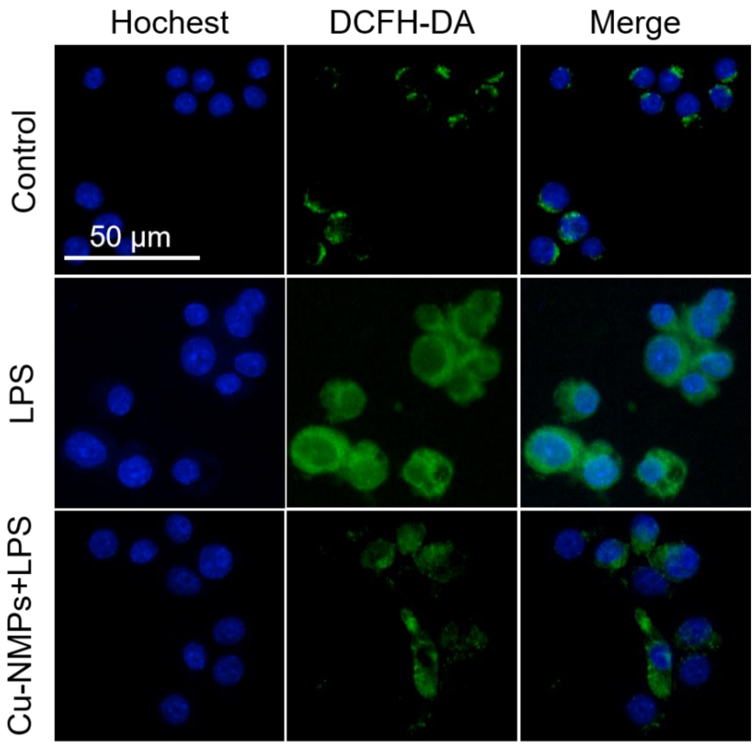

Figure S11. Related to **Figure 5.** Fluorescence microscopy images of ROS level in RAW264.7 with different treatments.

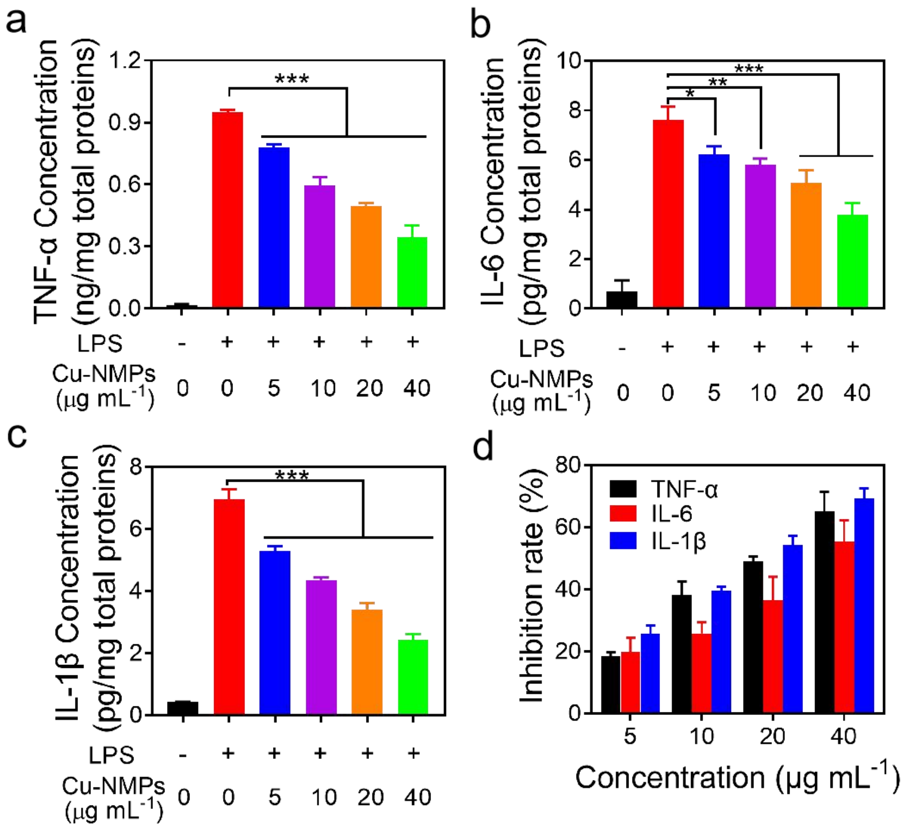

Figure S12. Related to **Figure 5.** The expression level of pro-inflammatory cytokines in LPS-stimulated RAW264.7 with different treatments. TNF-α (a), IL-6 (b) and IL-1β (c) expression levels in LPS-stimulated RAW264.7 with different concentrations of Cu-NMPs. (d) The inhibition rate of TNF-α, IL-6 and IL-1β expression with different concentrations of Cu-NMPs. Inhibition rate (%) = $[(C_p-C)/(C_p-C_n)] \times 100$ (C_n, C_p and C refer to the concentration of protein in negative control group (PBS treatment), positive control group (LPS treatment) and sample groups, respectively). Significance between each group was calculated using ANOVA with Tukey post hoc test. *P < 0.05, **P < 0.01, ***P < 0.001. Results are expressed as the mean ± S.D. of at least three independent experiments measured in triplicate.

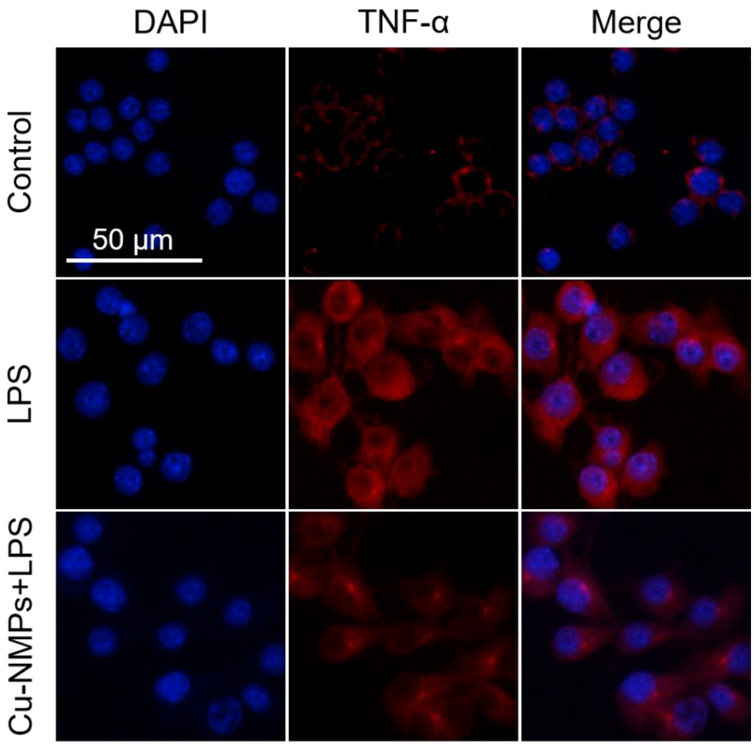

Figure S13. Related to **Figure 5.** Fluorescence microscopy images of TNF-α expression level in RAW264.7 with different treatments.

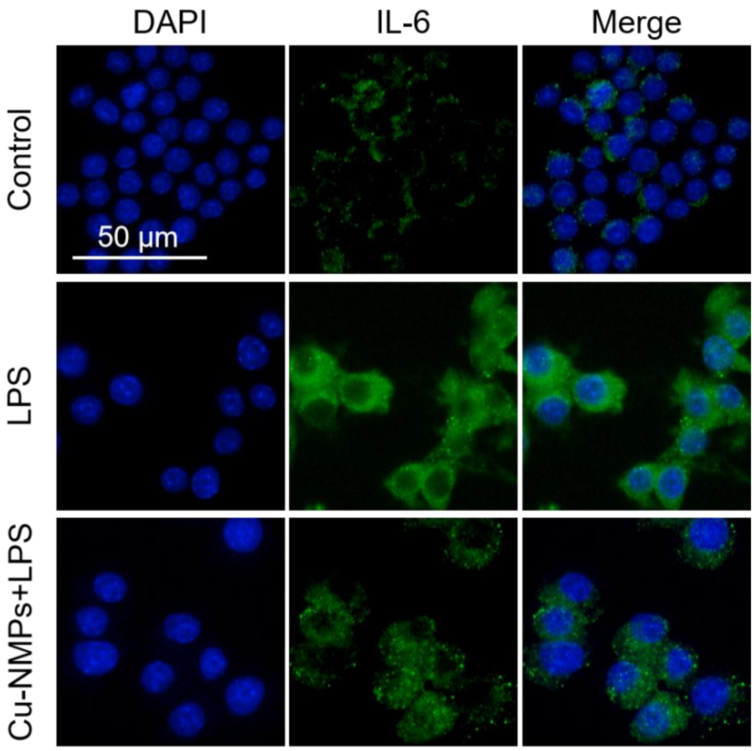

Figure S14. Related to **Figure 5.** Fluorescence microscopy images of IL-6 expression level in RAW264.7 with different treatments.

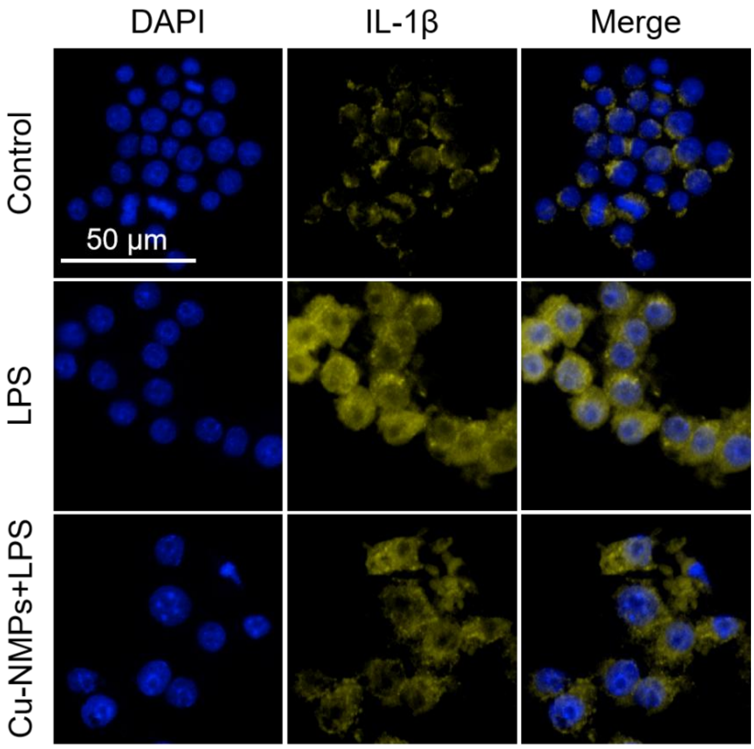

Figure S15. Related to **Figure 5.** Fluorescence microscopy images of IL-1β expression level in RAW264.7 with different treatments.

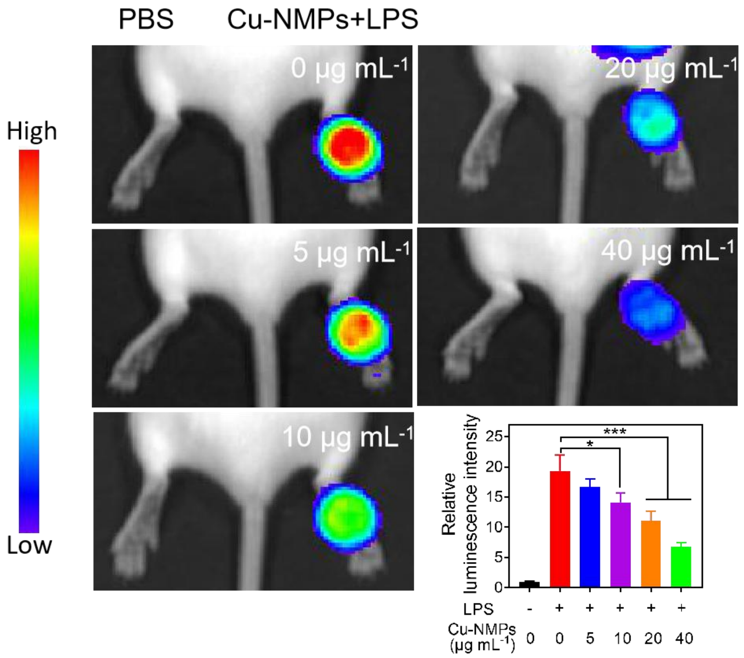

Figure S16. Related to **Figure 6.** *In vivo* bioluminescence images and corresponding luminescence intensities of ROS level in LPS-induced inflamed paws of mice with different concentrations of Cu-NMPs. Significance between each group was calculated using ANOVA with Tukey post hoc test. *P < 0.05, ***P < 0.001. Results are expressed as the mean ± S.D. of at least three independent experiments measured in triplicate.

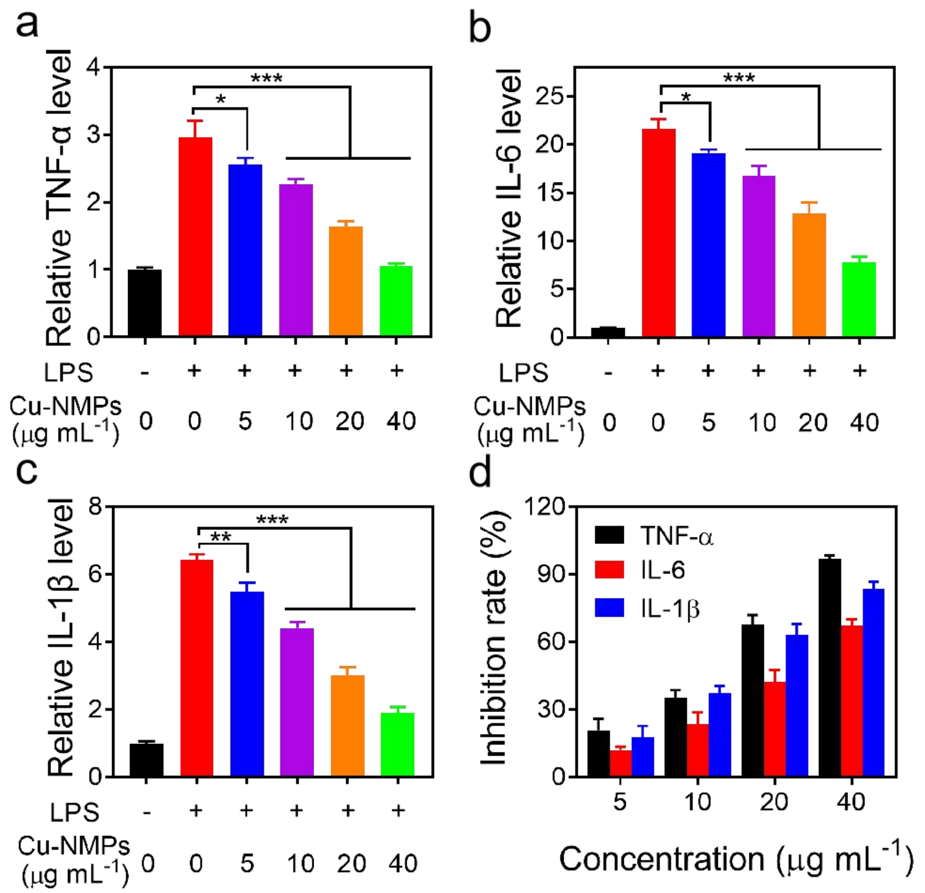

Figure S17. Related to **Figure 6.** The expression level of pro-inflammatory cytokines in LPS-induced inflamed paws with different treatment. TNF-α (a), IL-6 (b) and IL-1β (c) expression levels in LPS-induced inflamed paws of mice with different concentrations of Cu-NMPs. (d) The inhibition rate of TNF-α, IL-6 and IL-1β expression in LPS-induced inflamed paws of mice with different concentrations of Cu-NMPs. Inhibition rate (%) = $[(C_p-C)/(C_p-C_n)] \times 100$ (C_n, C_p and C refer to the concentration of protein in negative control group (PBS treatment), positive control group (LPS treatment) and sample groups, respectively). Significance between each group was calculated using ANOVA with Tukey post hoc test. *P < 0.05, **P < 0.01, ***P < 0.001. Results are expressed as the mean ± S.D. of at least three independent experiments measured in triplicate.

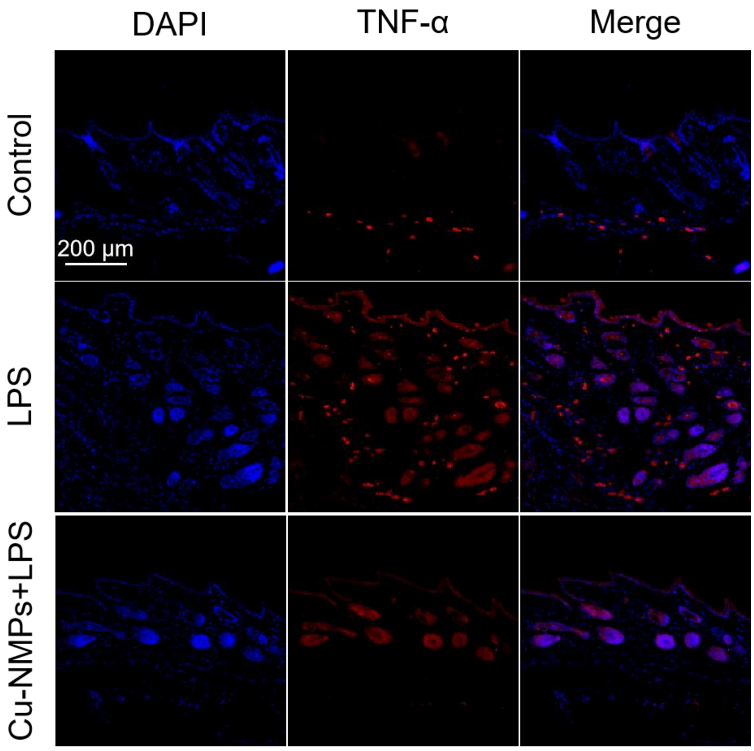

Figure S18. Related to **Figure 6.** Immunofluorescence staining of TNF-α expression in LPS-induced inflamed paws of mice with different treatment.

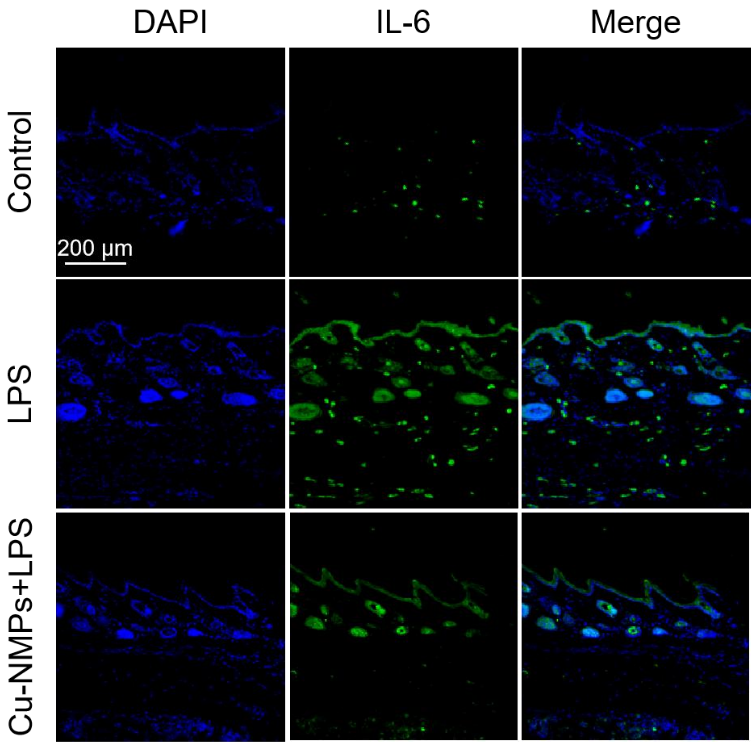

Figure S19. Related to **Figure 6.** Immunofluorescence staining of IL-6 expression in LPS-induced inflamed paws of mice with different treatment.

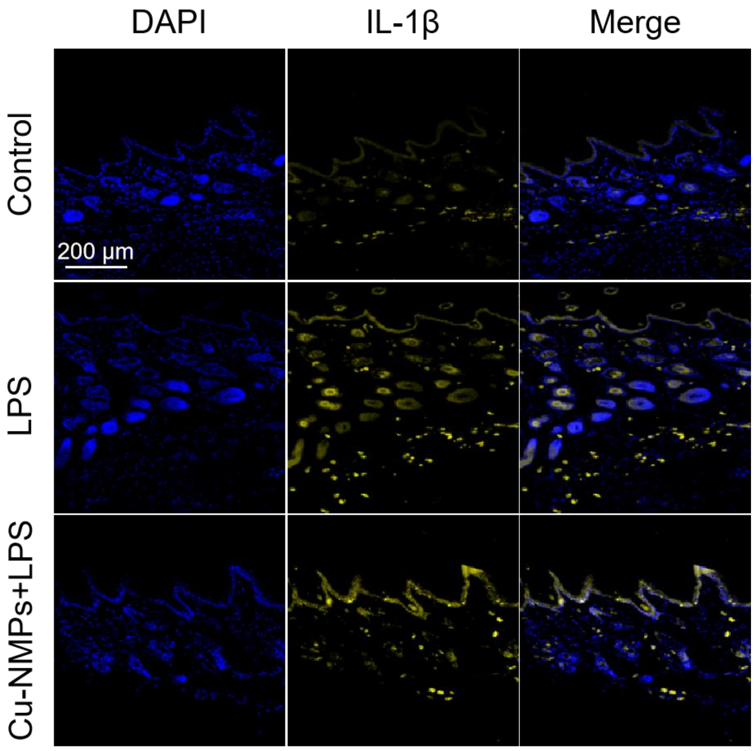

Figure S20. Related to **Figure 6.** Immunofluorescence staining of IL-1β expression in LPS-induced inflamed paws of mice with different treatment.

Supplemental Tables

Table S1. Related to **Figure 1.** The metal ions content in its corresponding M-NMPs.

Metal	Content (%)
Fe	4.0
Cu	5.1
Mn	4.2

Table S2. Related to **Figure 2.** The Michaelis-Menten constant (K_m) and maximum reaction rate (V_{max}) of Fe-NMPs and Cu-NMPs with TMB and H_2O_2 as the substrates for POD-like catalysis activity.

Catalyst	[E] (μg mL^{-1})	substrate	K_m (mM)	V_{max} (10^{-8} M s^{-1})
Fe-NMPs	10	TMB	0.46	2.49
Fe-NMPs	10	H_2O_2	394	4.11
Cu-NMPs	10	TMB	0.585	1.8
Cu-NMPs	10	H_2O_2	484	1.93
